# Resilience under the chilling effect: how social support and digital media reshape online political participation among Chinese youth

**DOI:** 10.3389/fpsyg.2025.1634604

**Published:** 2025-08-04

**Authors:** Jiamei Yang, Hao Jiang, Mingjiang Dai, Wodong Guo

**Affiliations:** ^1^Institute of Finance and Trade Economics, Sichuan Academy of Social Sciences, Chengdu, China; ^2^School of Journalism and Communication, Tsinghua University, Beijing, China; ^3^Institute of Journalism and Communication, Sichuan Academy of Social Sciences, Chengdu, China; ^4^School of Journalism and Communication, Sun Yat-sen University, Guangzhou, China

**Keywords:** chilling effect, fragmented authoritarianism, digital media use, online political participation, social support

## Abstract

This study examines how digital media use and perceived social support influence political participation among Chinese youth. We administered a large survey (*N = 6,855*) and employed structural equation modeling (SEM) to test a theoretical path model. Key measures included self-reported intensity of digital media use, multidimensional perceived social support, and online political participation. The model hypothesized that social support directly predicts online political participation and also indirectly affects participation via digital media use, with perceived state presence moderating the model. Results indicate that higher perceived social support significantly predicts greater digital media use and higher levels of political participation. Digital media use partially mediates the positive effect of social support on participation. Moreover, stronger perceptions of state monitoring amplified the positive relationship between media use and engagement, consistent with expectations from a fragmented authoritarian context. These findings suggest that robust social support from local community and active online engagement jointly sustain youth civic involvement even under restrictive conditions. The study contributes empirical evidence on the dual role of online platforms and social support in Chinese political socialization, with implications for enhancing civic resilience in fragmented authoritarian settings.

## 1 Introduction

Chinese youth today are permanently online and connected, inhabiting a broad spectrum of online communities from activist networks to online interest groups (Vorderer et al., 2016). In China’s mediated society, digital platforms have disrupted traditional media gatekeeping and now serve as crucial channels for continuous political information. Online environments provide unprecedented opportunities for youth engagement: on one hand, social media can facilitate diverse forms of participation and foster a kind of “internet democratic politics” virtuous cycle; on the other hand, the flood of politicized content can breed apathy, polarization, and disenchantment (Kruikemeier et al., 2014; Boulianne and Theocharis, 2020; Pang et al., 2022). These mixed trends underscore an urgent need to understand how Chinese digital natives use social media in context, and how their offline social surroundings translate into real-world civic action. In China’s specific context, offline protest is restricted, making online expression often the primary outlet for youth political voice. Political leaders have explicitly encouraged prosocial participation, emphasizing slogans like “When youth thrive, the nation thrives…” to rally young people as national stakeholders. Such leadership messages coincide with rising civic consciousness: recent surveys show steadily increasing youth involvement in both online and offline public affairs (Oser et al., 2022).

To analyze this dynamic, we draw on theoretical perspectives tailored to China’s political environment. *Fragmented authoritarianism* highlights that Chinese state power is dispersed across central and local levels (Lieberthal, 1992). In practice, local governments may sometimes accommodate or even support grassroots political activity, even as central authorities enforce strict media controls. Within this landscape, state presence – the pervasive awareness of potential surveillance and monitoring online – can induce a psychological *chilling effect* (Penney, 2019), causing individuals to self-censor. By contrast, social support from one’s offline network (the sense of being cared for and aided by family, friends, or community) is well known to motivate civic engagement. These frameworks together suggest that Chinese youths’ political participation will be shaped by both the deterrent effect of state oversight and the encouraging effect of social networks.

Building on this context, the present study examines how digital media use and perceived social support jointly shape political participation among Chinese youth. We propose and test a structural equation model and test the hypotheses in a large regional survey of youth, measuring online engagement, state presence, and levels of social support. This approach allows us to identify which forms and intensities of media use best translate offline support into actual civic action. Ultimately, the analysis aims to clarify how offline and online factors together sustain youth political involvement under China’s mediated, authoritarian conditions.

## 2 Literature review

### 2.1 Online political participation

Political participation is a form of behavior through which individuals attempt to influence government decision-making, and it also serves as a key indicator of democratic governance in contemporary societies. Since the twentieth century, the definition of political participation has transcended electoral participation (such as voting and campaigning) to encompass unconventional political behaviors (e.g., rallies and protests), and even non-political actions (e.g., social volunteering) have been included (Theocharis and van Deth, 2017). Some researchers have classified political participation behaviors into nine modes along an active-passive dimension, including voting, campaign activities, protest activities, contacting, collective participation, consumption, news attention, discussion, and forms of expression (Gibson and Cantijoch, 2013). However, it is noteworthy that while youth engage in politics through voting and other established forms of political participation (such as party membership or signing petitions), new forms of political participation that may not conform to traditional definitions—such as personal political podcasts, participatory theater, and many other “creative,” “personalized,” or “self-expressive” acts—are also enabling young people to participate in politics in their own ways, on their chosen political projects, and through the identity characteristics they wish to express (Chou et al., 2015; Marsh and Akram, 2015; Saud et al., 2020). The impact of multiple forms of political participation on social development is complex. Furthermore, the disconnection from traditional politics, critical attitudes, and even non-participation—political apathy—exhibited by young people are becoming increasingly prominent (Amnå and Ekman, 2014; Cammaerts et al., 2014).

The literature on Online Political Participation has not adequately addressed this categorization issue. The conventional approach is simply to distinguish “online” behaviors from “offline” ones. Clearly, expanding this concept gives rise to conceptual and methodological challenges (Theocharis and van Deth, 2017), because political participation should encompass only those actions explicitly directed at institutions or political processes, or those that have the potential to directly influence policy or the selection of policymakers. Some scholars have incorporated a wide range of activities into different metrics for online political participation (Hooghe et al., 2014). For instance, the act of seeking political information on platforms such as internet digital media has also been included in new models of online political participation (Jensen, 2013). Traditionally, seeking online political information offline is considered political communication; however, in online activities, it is difficult to clearly demarcate political participation from political communication because these activities are definitionally communicative. Without a more detailed definition of political motivation, online political participation as a new concept struggles to address this challenge. Research has found that citizens’ online and offline political participation can significantly predict each other (de Zuniga et al., 2012). Although online and offline political participation may appear to be opposing directions of engagement, both are indispensable components of political participatory behavior. The higher the frequency of online expression among digital media users, the more likely they are to transition from an observer to a participant when expressing views, ultimately leading to real-world political action (Gil de Zuniga et al., 2014). In summary, the concept of political participation has been adjusted and expanded due to changes in the social environment, and the emergence of digital media has further extended the boundaries of this concept by facilitating new forms of political action.

### 2.2 Digital media use

As the potential of information and communication technologies (ICT) to advance knowledge is increasingly realized, the development of electronic infrastructures has become a critical element of process-oriented research (Barjak et al., 2013). Research on digital media use indicates that political information obtained from different media types shapes audiences’ attitudes toward political systems in distinct ways. Some scholars argue that state-owned media content bolsters regime support, whereas political messages encountered on social platforms such as Weibo correlate negatively with regime support. Conversely, accessing political information via WeChat has been found to enhance nationalist sentiment, which in turn increases support for the political system (Wang and Kobayashi, 2021). Other researchers focusing on social participation among Hong Kong youth have identified media use as a mediator between psycho-social predictors of civic engagement and actual participation behaviors (Lee et al., 2017). Ultimately, Digital Media Use not only provides the public with a platform for self-expressive political engagement but also effectively facilitates connection, organization, and coordination among social actors (Foot and Schneider, 2002; Wattal et al., 2010).

Today, governments and professional planners increasingly employ digital media to solicit public opinion, disseminate information, and facilitate participatory planning practices; digital platforms have thus emerged as salient channels of political communication (Feezell, 2018; Popa et al., 2020). As principal carriers of political information, media, political participation, and social support are now recognized as interrelated domains warranting scholarly attention. The widespread adoption of ICT has progressively transformed both the intensity and nature of public participation, fostering novel forms of interaction between governments and citizens. Compared with traditional media (e.g., newspapers, television), new platform-based media are more readily embraced by users, who can engage in discussions or activities and receive real-time feedback (Lin and Kant, 2021). A robust linkage exists between digital media use and political behavior (Boulianne, 2018; Boulianne and Theocharis, 2020; Jiang and Kontauts, 2019; Pang et al., 2022), yet most studies conduct predominantly unidirectional analyses of media use and political actions, while investigations into the social support–media use–political participation pathway remain scarce. Prior research shows that participants in online and offline political activities differ, with youth relying more heavily on online engagement than on offline participation (Vitak et al., 2011), studies examining both modes of engagement within the same cohort are limited. Moreover, Online Political Participation has no direct analogue in the pre-digital era. Examples include publicly following political figures, posting written comments on behalf of others, sharing political news items (with or without commentary and social endorsement), organizing highly visible online petitions, and orchestrating collective protests within legally permissible frameworks (Theocharis and van Deth, 2017).

Regarding the relationship between digital media use and political participation, no research to date has convincingly revealed a causal link between the use of specific media and the acquisition of political participation information (Boukes, 2019). Some researchers argue that digital media exposure negatively affects political attitudes, positing that negative content in digital media can lead audiences to become disengaged from public affairs, cynical, and apathetic towards politics, thereby adversely impacting their political attitudes (Norris, 2001); that is, the use of digital media brought about by the internet reduces civic and political participation (Vitak et al., 2011). However, this viewpoint is contested. Some studies indicate that digital media exposure has a positive impact on attitudes toward political participation, suggesting that media exposure promotes favorable political attitudes (Kim and Hoewe, 2023). Furthermore, the relationship between media exposure and political attitudes can be influenced by other factors, such as technological factors (Boulianne and Theocharis, 2020). Although most countries have not yet implemented electronic voting, significant differences already exist between online and offline political participation (Ruess et al., 2023).

As noted above, digital media constitute a key source of social support and political participation. Beyond distinctions among media types, research into digital media’s impact on civic engagement must distinguish among varying information-seeking motivations. Citizens increasingly obtain news via online channels (Welbers and Opgenhaffen, 2019; Dvir-Gvirsman and Tsuriel, 2022), and they also use smartphone messaging applications to communicate with peers or to upload user-generated content (Serfass and Sherman, 2015; Su and Xiao, 2021; Berrocal-Gonzalo et al., 2023). Early studies have already established a substantial relationship between Internet use (Boulianne, 2009) or social media use (Boulianne, 2015, 2018) and political behavior. Carpini (2000) found that the more frequently digital media are used, the more likely political alienation becomes, leading to reduced time spent on public affairs. Dashti et al. (2015) further noted that social media, in fact, does not encourage expression. By this stage, the chilling effect extends far beyond real-world self-censorship driven by fear among digital citizens to include online behavioral adjustments made in response to privacy concerns and data surveillance. In light of this, the relationship between Digital Media Use and Online Political Participation remains an open question that warrants further investigation.

### 2.3 Fragmented authoritarianism: the constraints and enablers facing online political participation

The Internet has dismantled the State’s monopoly over mass media (Castells, 2012), thereby challenging political authorities’ control over citizen engagement. In response, the Chinese government has sought to regulate domestic information flows by adapting its control mechanisms—primarily through enhanced Internet censorship (King et al., 2013), innovative State propaganda paradigms, and promotion of multi-stakeholder participation in Internet governance (Shen, 2016). These mechanisms are generally understood to serve core State objectives—such as agenda setting and shaping public perceptions (Brady, 2009; Chen and Yang, 2019), influencing attitudes and opinions (Pan et al., 2022), and raising the costs of citizen protest and collective action (King et al., 2013). However, in the concrete practices of media censorship and Internet governance, the central government’s mandate to maintain social stability is refracted through the interests and capacities of local agencies. This has led to divergences—and at times conflicts—in the bureaucratic state’s modes of governance over civil society.

Fragmented Authoritarianism (Lieberthal, 1992, pp. 8–9) insightfully explains the internal dispersion of State power. Scholars contend that a defining feature of Chinese politics is the disconnection in decision-making below the apex of power, marked by “profound jurisdictional fragmentation” among bureaucratic agencies (Mertha, 2005, p. 27). Such horizontal fragmentation can create opportunities for alliances between local governments and civil society in the policy-entrepreneurship process. Accordingly, this paper adopts the theoretical perspective of Fragmented Authoritarianism to analyze how State/government power affects political participation. Two intermediate concepts are employed to elucidate this complex and uncertain process: the chilling effect and Social Support.

#### 2.3.1 State presence: the chilling effect in digital spaces

The chilling effect refers to individuals’ anticipatory self-restraint of speech and behavioral freedoms due to perceived surveillance or fear of rule violations (White and Zimbardo, 1980). At the psychological level, this concept explains how State power induces “self-restraint” in political participation rather than overtly suppressing civil society. The literature in law, communication studies, and social media research focuses on the chilling effect within the contexts of citizen privacy and surveillance. With the advancement of new media technologies, issues of privacy, surveillance, and speech control have become increasingly salient in the regulation of broadcast, television, and the Internet. Studies have explored the insights and risks of integrating these issues with chilling-effect theory, prompting a reexamination of its theoretical implications. Individuals whose privacy is threatened in specific contexts may fear full self-expression, thereby triggering the chilling effect (Nissenbaum, 2004). Exploration of the relationship between context and privacy expectations highlights the interaction between privacy and the chilling effect (Nissenbaum, 2004).

While most studies focus on how the Chinese government and corporations rigorously censor social media through firewalls, sensitive-word filters, and multi-layered network surveillance, they have yet to address the resultant chilling effect (Ruan et al., 2021). In this article, I follow Penney’s (2019) collectivist paradigm of the chilling effect to examine how State Presence influences self-limitation in civic political participation, including collective action, decision-making, group knowledge, and the right to information.

#### 2.3.2 Policy entrepreneurship in democratic reform: local community’s social support for online political participation

The decentralized control characteristic of authoritarianism does not entirely dismantle civil society. On the contrary, it can introduce uncertainty for political elites and entice local governments to engage in “entrepreneurial censorship,” a social support system characterized by a blend of democratic experimentation and entrepreneurial initiatives. Provinces and municipalities such as Guangdong, Shanghai, and Fujian—economically advanced regions and political reform pilots—have in recent years demonstrated an attitude and practices of accommodating, guiding, and even collaborating with civic movements and collective protest events, providing empirical support for this argument.

Theoretically, Social support refers to the perception among individuals in social interactions that they are cared for, loved, and respected, thereby alleviating stress arising from factors such as illness (Cobb, 1976). It also encompasses instrumental assistance, namely the exchange of resources or mutual aid between individuals (Hupcey, 1998; Oh et al., 2013). Informational and instrumental components of social support are conveyed, perceived, and received through social interaction (Frison and Eggermont, 2015). In interpersonal contexts, social support is defined as the provision of tangible assistance to individuals and their embedding within a network perceived as caring, loving, and readily accessible in times of need (Kaniasty et al., 2020). Social support typically comprises emotional concern, instrumental help, and informational appraisal (Brunstein et al., 1996). Factors influencing social support include the nature of social ties or networks, recipients’ perceptions of available support, and the personalities of both providers and recipients (Razurel and Kaiser, 2015). The emotional care, attention, and assistance individuals perceive constitute their sense of social support, which is a critical factor for most people living within social structures (Vedder et al., 2005).

Classifications of social support follow traditional multidimensional frameworks. Core dimensions include informational support, emotional support, and tangible support (Kim et al., 2012; Phua, 2013; Rui et al., 2013). Some studies have also identified other classificatory dimensions of social support, including emotional support, tangible support, informational support, group identity, and respect (Cutrona and Suhr, 1992); dimensions of emotion, information, tangibility, and respect (Braithwaite et al., 1999; Oh et al., 2013); tangible support, emotional support, informational support, and peer support (Cohen, 2004); and emotional support, informational support, tangible support, respect support, and advice (Dai, 2018), among others. Relevant research commonly adopts the classic social support classification that includes informational support, emotional support, and tangible support. Based on the subjects of this study, the aforementioned three-type classification framework is adopted. Among these, informational support involves providing information and advice based on facts, definitions, or data to help others solve problems; emotional support comprises behaviors or information that generate intimacy, trust, and a sense of belonging within a community; and tangible support is the provision of direct services or material assistance (Dai, 2018). In recent research, perceived social support generally refers to the psychological and material support or aid that individuals receive from various aspects of their society within their social environment. Some scholars have pointed out that, compared to other media, individuals can obtain more social support through social media. Research investigating the relationship between adolescent social support and media use via new media platforms such as Facebook, Weibo, and WeChat indicates that the internet is becoming an important source of social support (Shi and Chen, 2014). The use of social media not only has a direct impact on the quality of real life but also exerts an indirect influence through social support. Young people who receive a higher degree of real-life social support are more inclined to proactively use social media tools to construct their self-image, thereby further optimizing their real-life social relationships (Wright et al., 2013).

Based on the literature review, we conceptualize a theoretical model in which (1) Social Support exerts a direct effect on Online Political Participation, (2) Social Support influences Digital Media Use and thereby indirectly affects political participation, (3) Digital Media Use affects Online Political Participation, and (4) State Presence moderates the effect of Digital Media Use on Online Political Participation. To test whether State Presence moderates the relationship between Digital Media Use and Online Political Participation among youth, we propose the following hypotheses:

*H1*: Perceived Social Support among young people is positively correlated with their Online Political Participation.

*H2*: Digital Media Use mediates the relationship between perceived Social Support and Online Political Participation among young people.

*H2a*: Digital Media Use among young people is significantly and positively correlated with their Online Political Participation.

*H2b*: Digital media use among young people shows a significant positive correlation with their political participation behavior.

*H3*: Perceived State Presence moderates the relationship between Digital Media Use and Online Political Participation among young people. Specifically, the higher the perceived level of State Presence, the stronger the positive effect of Digital Media Use on Online Political Participation.

**Figure 1:**
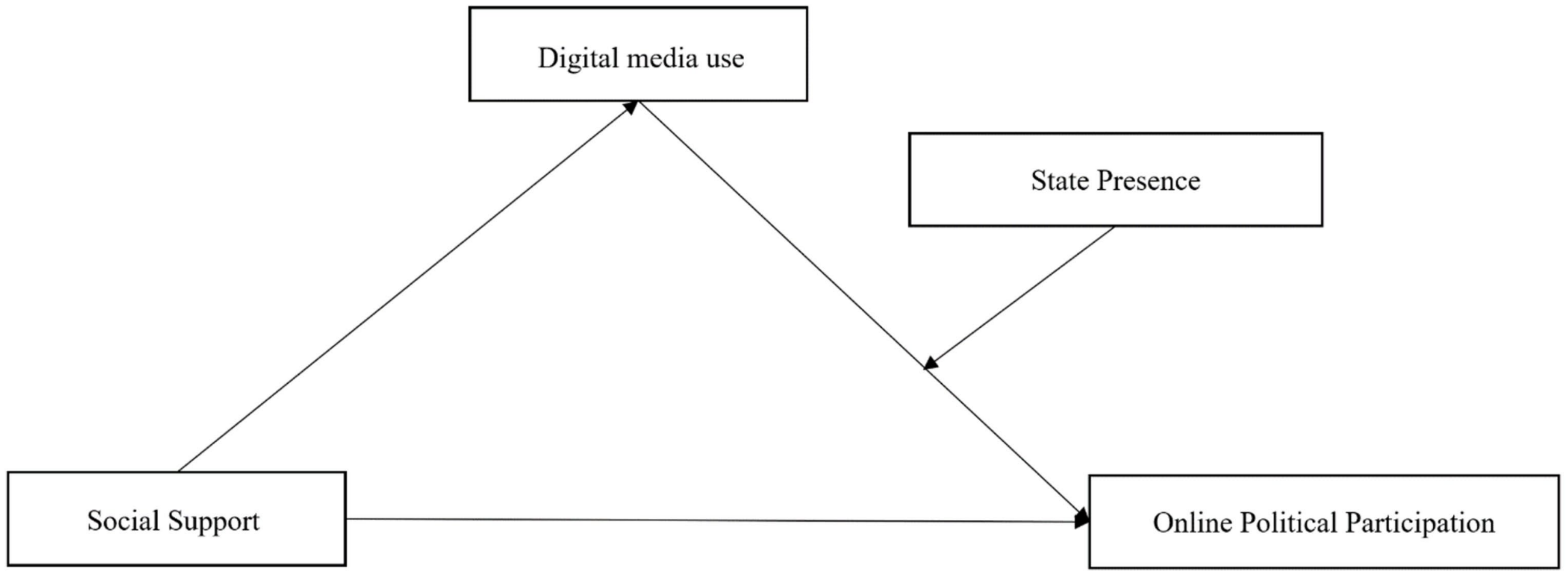
Research framework.

We will test a path model encompassing all the above hypotheses (see Figure 1). In particular, the model examines the interrelations among perceived Social Support, Digital Media Use, State Presence, and Online Political Participation. In summary, the objective of this study is to determine whether young people seek more real-world social support in political participation, to what extent digital media use strengthens/weakens this relationship, and how State Presence influences the mechanism by which Digital Media Use affects Online Political Participation.

## 3 Materials and methods

The scales used in the questionnaire are provided in the Supplementary material, along with the informed consent form approved by the institutional ethics committee. Following approval by the University Institutional Review Board, this study conducted an online questionnaire survey via Tencent Questionnaire from August to November 2018, targeting youths aged 18–40 in Guangdong Province, a coastal region of China. All participants provided written informed consent prior to participation, confirming their agreement with the study protocols.

The survey specifically examined youths’ perceived social support, digital media engagement, and political participation behaviors. A total of 6,855 valid questionnaires were collected, including 2,934 males (42.8%) and 3,921 females (57.2%). The age distribution comprised 3,923 respondents (57.2%) aged 18–24, 1,412 (20.6%) aged 25–29, and 1,520 (22.2%) aged 30–40. Regarding the age-range definition for youth, this study adopted a hybrid criterion: while referencing the WHO broad definition of youth (15–44 years), we focused on the 18–40-year cohort to align with legal adulthood threshold (≥18 years) and regional research priorities on young adult civic engagement (Omar et al., 2016).

This study employs SPSS 27.0 and PROCESS 5.0 for statistical analysis and reliability–validity testing. We first conduct factor analysis in SPSS to confirm that the hypothesized item–variable relationships hold in our sample; we then specify the mediation and moderation models using the PROCESS macro; following model estimation, fit indices were assessed, and the bootstrap method was employed to calculate confidence intervals for indirect effects to determine the presence of mediation.

## 4 Measurement

### 4.1 Perceived social support

This study adopted the Multidimensional Scale of Perceived Social Support (MSPSS) (Zimet et al., 1988; Osman et al., 2014) and adapting it to the current context of perceived social support among Chinese youth, this study incorporated supplementary scales measuring infrastructure, policy support, social services, social environment, and well-being. Ten social support items were selected as outcome variables: “Basic Public Infrastructure,” “Governmental Policy Support,” “Social Services,” “Growth Environment,” “Family,” “Community,” “Social Environment,” and “Well-being.” A five-point Likert scale (scored *1* through *5*) was used, with respondents evaluating their satisfaction with various factors of the youth development environment as: “very dissatisfied,” “dissatisfied,” “neutral,” “satisfied,” and “very satisfied.” For regression analysis requirements, this scale was reverse-scored such that higher scores indicate greater perceived social support. The scale’s *Cronbach’s α* coefficient was *0.953*.

### 4.2 Digital media use

Information-seeking behaviors and the usage frequency of digital media applications served as explanatory variables. Information-seeking behaviors encompassed: (1) following local policy announcements via digital media, (2) monitoring local social development through digital media, (3) accessing online resources, and (4) following news events through digital media. The digital media categories included: (1) WeChat (Social media platform); (2) Sina Weibo (Blog platform); (3) Zhihu (Online Q&A communities); (4) news platforms such as Toutiao; (5) short-video social platforms such as Douyin and Kuaishou; (6) video-sharing websites such as Youku and Bilibili; and (7) audio platforms such as Ximalaya, covering the primary digital media types used by Chinese youth. Responses were recorded on a 5-point Likert scale: “Never” (1), “Seldom” (2), “Occasionally” (3), “Sometimes” (4), and “Frequently” (5), corresponding to the frequency of both information-seeking behaviors and app usage. The scale’s *Cronbach’s α* coefficient was *0.849*.

### 4.3 State presence

The level of State Presence was measured across dimensions including (1) awareness of national ideology, (2) support for state ideological dissemination, and (3) support for enhanced state ideological regulation. Aligned with Chinese youths’ understanding of State Presence, five items gauging the level of agreement were used as the outcome variable: “Exposure to and endorsement of national ideology,” “State participation in internet information governance,” “State role in constructing unified values,” and “State enhancement of ideological propaganda.” Agreement levels were measured on a 5-point Likert scale: “Strongly Disagree” (1), “Disagree” (2), “Neutral” (3), “Agree” (4), and “Strongly Agree” (5). The scale’s *Cronbach’s α* coefficient was *0.952*.

### 4.4 Online political participation behavior

The scale developed by Wang et al. (2019) was adapted for assessment. The original scale comprises seven items inquiring about engagement in activities like online voting, online petitioning, and online boycotts, categorizing youth online political participation into three dimensions: Expressive participation, Online Activism, and Online Petition. Adapting to the specific context of Chinese youth political engagement, this study measured the frequency of 12 behaviors as the outcome variable: “Publicly sharing information related to public affairs,” “Organizing collective action and protest on social issues,” “Online donating (Jiao et al., 2021),” “Following policy announcements,” and “Participating in online discussions” (see Table 1 for the full scale). Frequency of participation was measured on a 5-point Likert scale: “Never” (1), “Seldom” (2), “Occasionally” (3), “Sometimes” (4), “Frequently” (5). The overall scale for Online Political Participation demonstrated excellent reliability (Cronbach’s α = *0.940*), with subscales also showing good reliability: Expressive Participation (Cronbach’s α = *0.936*), Online Activism (Cronbach’s α = *0.830*), Online Petition (Cronbach’s α = *0.862*).

**Table 1:** Measurement items and reliability and validity tests for online political participation types.

Behavioral pattern	Item	Factor loading	KMO	Cronbach’s α
Expressive participation	Comment on news articles/news portal websites online	0.893	0.857	0.936
	Publicly express opinions or publish information about social/public affairs online	0.932		
	Repost text, videos, and images related to social/public affairs online	0.907		
	Participate in online discussions about social/public affairs	0.930		
Online activism	Post online (e.g., on Weibo/social media) seeking help when encountering difficulties or social injustice	0.913	0.741	0.830
	Seek help from social media influencers when encountering difficulties or injustice	0.915		
	Organize or participate in collective actions or protests concerning social/public affairs	0.735		
	Participate in actions like online donations	0.680		
Online petition	Contact government departments regarding social/public affairs	0.842	0.791	0.862
	Provide feedback to People’s Congress deputies or CPPCC members concerning social/public affairs	0.912		
	Contact newspapers, radio stations, or TV stations regarding social/public affairs	0.895		
	Join groups or online communities related to social/public affairs	0.716		

### 4.5 Demographic variables

The individual-level demographic variables employed in this study include gender, age, educational attainment, only-child status, household registration type (hukou), average monthly personal income, occupation (i.e., cadre of party or government agencies, business manager, corporate employee, professional/technical staff, worker, farmer), marital status, and living arrangement.

## 5 Results

### 5.1 Descriptive statistics

A total of *6,855* questionnaires were collected; *25* invalid responses were excluded, yielding *6,830* valid cases. The sample comprised *42.7%* males and *57.3%* females; over half of respondents were aged *18–24*, and those under *30* accounted for *77.9%* of the total, indicating a generally youthful age profile. Regarding educational attainment, *89.2%* of respondents had received education at or above the high school level, suggesting a relatively high level of educational achievement among the youth sample. In terms of hukou registration, the proportion of respondents with local (urban or rural) registration was roughly equal to those with non-local registration; local hukou accounted for *54%*, slightly higher than the *46%* holding non-local hukou. With respect to marital status, the majority of respondents were unmarried, comprising *71.9%* of the sample. Employment data revealed that many respondents were university students or early-career researchers, largely reflecting their education-appropriate age; in addition, a substantial proportion were employed by non-public enterprises (*27.7%*), followed by those in state-owned or state-controlled enterprises (*9.6%*), professionals and technical personnel (*8.6%*), and public sector employees, including civil servants (*7.4%*). Among the study participants (*N* = 6,830), Table 2 reports the means and standard deviations of the key research variables, which form the basis for the preliminary analyses presented below.

**Table 2:** Descriptive statistics of key research variables (mean and standard deviation).

Variable	Dimension	*M*	SD
Social support (*M* = 2.88, SD = 0.01)	Public infrastructure	3.03	0.01
	Policy support	3.04	0.01
	Social services	3.05	0.01
	Environmental security	3.10	0.01
	Family environment	3.27	0.01
	Community and neighborhood	3.22	0.01
	Well-being	2.13	0.01
	Socio-environmental perception	2.68	0.01
Digital media use (*M* = 2.50, SD = 0.01)	Information acquisition	3.17	0.01
	Usage frequency	1.83	0.02
State presence	---	3.33	0.01
Online political participation (*M* = 2.30, SD = 0.01)	Expressive participation	2.08	0.02
	Online activism	2.31	0.02
	Online petition	2.25	0.02

First, in terms of Social Support, youths’ overall satisfaction with real-world Social Support was moderate (*M* = 2.88, SD = *0.01*). Among the dimensions, “Family Environment” scored highest (*M* = 3.27, SD = *0.01*), indicating greater satisfaction with familial harmony compared to other forms of support. Satisfaction with “Community and Neighborhood” (*M* = 3.22, SD = *0.01*) also exceeded the overall mean. By contrast, satisfaction scores for “Public Infrastructure” (*M* = 3.04, SD = *0.01*), “Policy Support” (*M* = 3.04, SD = *0.01*), “Social Services” (*M* = 3.05, SD = *0.01*), and “Environmental Safety” (*M* = 3.10, SD = *0.01*) fell below the overall average, revealing a less optimistic outlook for these support dimensions. Notably, levels of Well-Being (*M* = 2.12, SD = *0.01*) and perception of the Social Environment (*M* = 2.68, SD = *0.01*) were comparatively low among youth.

In terms of Digital Media Use, youths’ information-seeking behaviors were generally high (*M* = 3.17, SD = 0.01). youths tended to spend no more than 1 h per day on each platform. According to the data, they showed stronger usage intentions for Sina Weibo (*M* = *3.12*, *SD* = *0.01*) and long-form video platforms (*M* = *2.96*, *SD* = *0.01*) compared with news platforms, short-video social platforms, audio platforms, and Zhihu, toward which their usage intentions were weaker.

Regarding perceived State Presence, overall levels were high. The item “I frequently encounter state-led ideological messaging” was especially prominent (*M* = 3.29, SD = *0.01*), indicating stronger recognition of ideological outreach than other dimensions. Ratings for “The State should strengthen Internet information governance” (*M* = 3.41, SD = *0.01*) and “The State should promote mainstream ideology online” (*M* = 3.42, SD = *0.01*) also significantly exceeded the overall mean for State Presence perception, reflecting robust support for online governance and ideological dissemination. However, the item “I am well-versed in the content and core tenets of socialist core values” received a lower score (*M* = 3.16, SD = *0.01*), falling below the overall average.

For Online Political Participation, overall engagement was below the midpoint (*M* = 2.30, SD = *0.01*), corresponding to “Rarely” to “Occasionally.” Among participation types, Online Activism was highest (*M* = 2.31, SD = *0.02*), followed by Online Petition (*M* = 2.25, SD = *0.02*) and expressive participation (*M* = 2.08, SD = *0.02*). Youth thus prefer activities such as posting on social media, seeking influencer support, and organizing collective protests, rather than engaging in online discussions of public affairs or contacting authorities and media.

Table 3 presents means, kurtosis, skewness, and intercorrelations of key variables. All skewness and kurtosis values met normality criteria, and no multicollinearity was detected. Correlation and VIF analyses showed no indicators exceeding thresholds (Campbell, 1960). Perceived Social Support correlated with Online Political Participation (*r* = 0.201, *p* < 0.05) and Digital Media Use (*r* = 0.427, *p* < 0.05), while Digital Media Use correlated with Online Political Participation (*r* = 0.608, *p* < 0.05). These findings provide preliminary support for H1, H2a, and H2b, although confirmatory structural equation modeling is required.

**Table 3:** Means, standard deviations, kurtosis, skewness, and intercorrelations of key study variables (*N* = 6,830).

Variables	Mean	Std. dev	Skewness	Kurtosis	1	2	3	4
Social support	2.88	0.01	0.17	−0.68	1			
Digital media use	2.50	0.01	0.27	−0.073	0.427**	1		
State presence	3.33	0.01	0.03	−1.01	0.717**	0.482**	1	
Online political participation	2.30	0.01	0.60	−0.29	0.201**	0.608**	0.153**	1

** Correlations are significant at the 0.01 level (two-tailed). *** After testing for multicollinearity, the VIF indices for all variables are below the threshold, indicating no multicollinearity issues among the variables.

### 5.2 Structural equation modeling

This study used Structural Equation Modeling (SEM) to test the proposed structural model. A minimum ratio of 10 observations per free parameter—resulting in a critical sample size of *200*—is required to achieve adequate statistical power for SEM (Hoe, 2008). With *N* = *6,830*, this study exceeds that requirement. To address the large sample size and potential distributional issues, we employed the bootstrap method to derive estimates from the existing data. Bootstrap involves repeated resampling with replacement to generate empirical samples for constructing confidence intervals (95% CI). As a nonparametric resampling procedure, it effectively mitigates nonnormality concerns. Prior research indicates that bootstrap procedures offer greater statistical power than the Sobel test and other sequential methods (Fritz and MacKinnon, 2007).

We first report model fit for the moderated mediation framework. Structural equation modeling was conducted using the PROCESS macro. Given the large sample size (*N* > 5,000), chi-square criteria are inappropriate; therefore, we selected fit indices less sensitive to N. After model correction, all indices indicated acceptable fit. Path estimates and test results are presented in two parts: the mediation model of Digital Media Use without State Presence as moderator (Table 4), and the moderated mediation model including State Presence (Table 5).

**Table 4:** Path estimates for the mediation model of digital media use (*N* = 6,830).

Dependent variable: Digital media use	Standardized	SE	*p*-value	LLCI	ULCI
Social support	0.427	0.011	<0.001	0.406	0.449
*R*^2^	0.183				
*F*	1530.575***				
Dependent variable: Online political participation					
Social support	−0.072	0.011	<0.001	0.093	0.052
Digital media use	0.639	0.011	<0.001	0.618	0.660
*R*^2^	0.374				
*F*	2045.740***				

**Table 5:** Path estimates for the moderated mediation model with state presence (*N* = 6,830).

Dependent variable: Digital media use	Standardized	SE	*p*-value	LLCI	ULCI
Social support	0.427	0.011	<0.001	0.406	0.449
*R*^2^	0.183				
*F*	1530.575***				
Dependent variable: Online political participation					
Social support	0.072	0.014	<0.001	0.046	0.099
Digital media use	0.686	0.011	<0.001	0.665	0.708
State presence	−0.228	0.014	<0.001	−0.256	−0.201
Digital media use × State presence	0.020	0.009	0.022	0.003	0.038
*R*^2^	0.398				
*F*	1133.034***				

To assess the indirect effect of Social Support on Online Political Participation via Digital Media Use, we applied the causal steps approach to test the mediation of Digital Media Use; results are presented in Table 4. The path from Social Support to Digital Media Use was significant (*β* = 0.427, *p* < 0.05), supporting H2a. Likewise, the path from Digital Media Use to Online Political Participation was significant (*β* = 0.639, *p* < 0.05), supporting H2b. The direct effect of Social Support on Online Political Participation was *β* = −0.072 (*p* < 0.05), indicating that Digital Media Use partially mediates this relationship and thus supporting H2. Notably, the direct effect of Social Support on Online Political Participation was negative, suggesting that higher perceived support was associated with lower participation when Digital Media Use is included—an inconsistency with our theoretical expectations that merits further analysis.

Hypothesis H3 posited that perceived State Presence moderates the effect of Digital Media Use on Online Political Participation. After standardizing the variables, we conducted moderation analysis; results are detailed in Table 5. The main effect of perceived State Presence on Online Political Participation was *β* = −0.228 (*p* < 0.05), indicating that higher State Presence perception suppresses participation. The interaction between Digital Media Use and State Presence was significant and positive (*β* = 0.020, *p* < 0.05). Both perceived Social Support and Digital Media Use exerted significant positive main effects on Online Political Participation. Thus, perceived State Presence positively moderates the Digital Media Use–Online Political Participation relationship: higher State Presence strengthens the positive effect of Digital Media Use, confirming H3.

**Figure 2:**
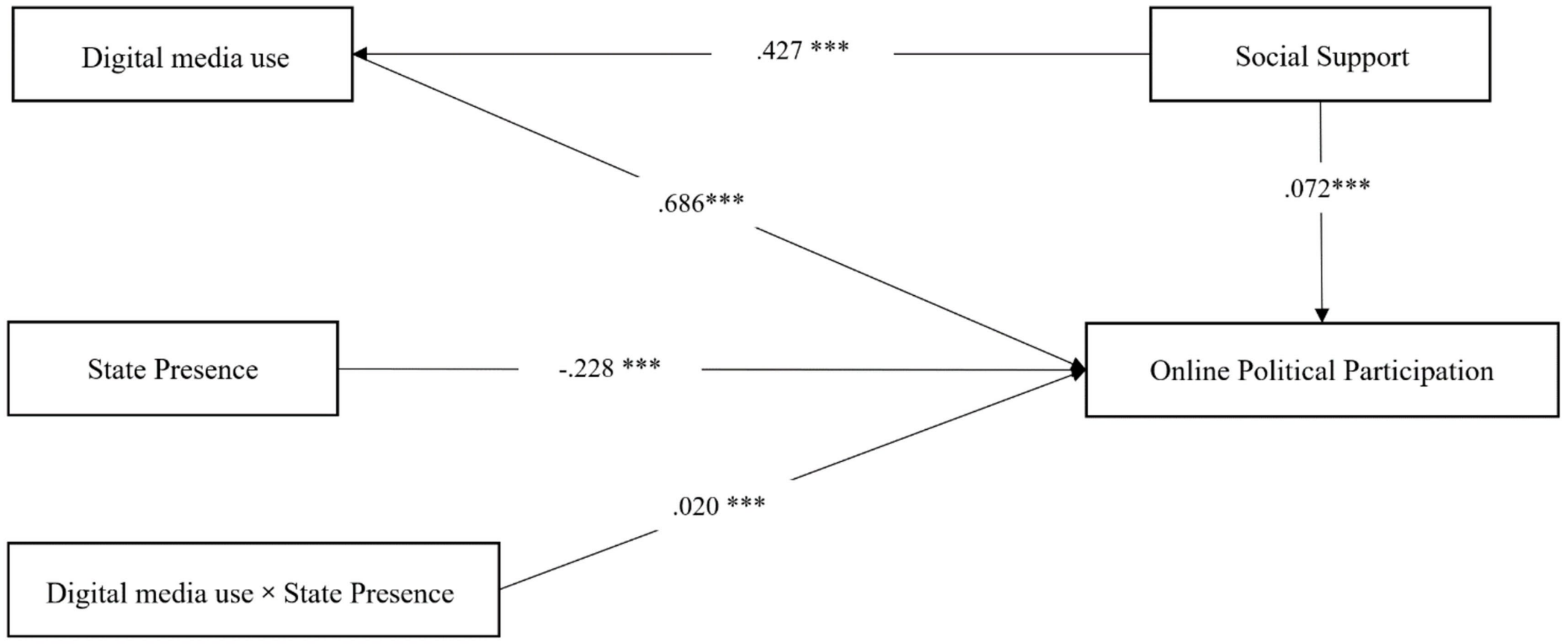
Structural model of Chinese youths’ perceived social support, digital media use, state presence and online political participation behavior.

As shown in Tables 4, 5, all key model-fit indices and latent-variable path coefficients were significant. After confirming acceptable model fit, we tested the proposed hypotheses. The results indicate good fit between the structural model and the data; the full model is depicted in Figure 2.

To examine whether perceived State Presence moderates the indirect effect of Social Support on Online Political Participation via Digital Media Use, we conducted a bootstrap analysis. When State Presence was included as a moderator, its moderating effect was significant (*β* = 0.020, *p* = 0.022), with a moderated mediation index of 0.009 (95% CI [0.001, 0.017]). This indicates that higher levels of perceived State Presence strengthen the indirect effect of Social Support on Online Political Participation through Digital Media Use.

To probe the boundary conditions of State Presence effects, we estimated the indirect effect of Digital Media Use at three levels of perceived State Presence (M − 1 SD, M, M + 1 SD) using PROCESS. As shown in Table 6, the mediation effect was 0.304 (95% CI [0.283, 0.326]) at high State Presence (M + 1 SD), compared with 0.292 (95% CI [0.274, 0.312]) at the mean and 0.284 (95% CI [0.264, 0.305]) at low State Presence (M − 1 SD). These results reveal an empowering effect of State Presence: when youth perceive stronger State Presence, the conversion efficiency of Digital Media Use into political participation increases by 4.1%. Thus, stronger perception of State Presence amplifies the indirect effect of Social Support on Online Political Participation via Digital Media Use.

**Table 6:** Moderated mediation effects of perceived state presence.

Social support → Digital media use → Online political participation			
State presence	Effect value	Boot LL 95% CI	Boot UL 95% CI
M-1SD	0.284	0.264	0.305
*M*	0.292	0.274	0.312
M + 1SD	0.304	0.283	0.326

In this section, we conduct focused tests and analyses of the study’s hypotheses and present the corresponding findings. A summary of the hypothesis verification results is provided in Table 7.

**Table 7:** Hypothesis testing summary.

Hypothesis ID	Hypothesis description	Test results
H1	Perceived Social Support among young people is positively correlated with their Online Political Participation.	True
H2	Digital Media Use mediates the relationship between perceived Social Support and Online Political Participation among young people.	True
H2a	Digital Media Use among young people is significantly and positively correlated with their Online Political Participation.	True
H2b	Digital media use among young people shows a significant positive correlation with their political participation behavior.	True
H3	Perceived State Presence moderates the relationship between Digital Media Use and Online Political Participation among young people. Specifically, the higher the perceived level of State Presence, the stronger the positive effect of Digital Media Use on online Political Participation.	True

## 6 Discussion

Our analysis reveals that social support, digital media use, and online political participation are tightly linked in China’s youth civic ecology. The structural equation modeling shows that perceived social support (from peers, family, or online communities) significantly predicts digital media use, which in turn drives online political participation. In other words, young people with stronger networks of encouragement and shared information use social media more intensively, and this mediates the effect of social support on their political engagement. We measured online participation in a multidimensional typology that distinguishes *expressive participation* (e.g., posting or commenting to express opinions), *online activism* (e.g., joining campaigns or mobilizing peers), and *online petitioning* (e.g., signing or organizing petitions). In our data, all three forms were associated with social support via media use.

Crucially, state presence perceptions significantly moderate these relationships. When youth report a high level of perceived *state presence* or monitoring online, overall participation tends to decline, consistent with a “chilling effect” of authoritarian control. In other words, awareness of government scrutiny weakens the motivating power of social support: even well-supported individuals participate less when they fear state surveillance. This chilling effect echoes findings from surveillance studies (e.g., Penney, 2016) showing that publicity about government monitoring can produce immediate and sustained drops in online activity. However, our results also surface a paradoxical nuance: under very high social support, increased state presence can actually *boost* online activism. In this subgroup, peers’ encouragement appears to embolden youths to push back, so state monitoring triggers counter-mobilization rather than retreat. This suggests that *fragmented authoritarianism* in China can yield unpredictable outcomes.

Based on the above conclusions, an important theoretical argument of this paper is that the chilling effect in China’s political communication should be situated within the context of Fragmented Authoritarianism. As Penney observes, “The chilling effect typically arises from ambiguous and uncertain contexts, such as vagueness in the law or an individual’s awareness that they may be monitored by the government or by peers on social media.” Furthermore, unlike propaganda and control in the ideological domain, the state’s censorship and governance of the Internet are not grounded in a “command-and-control” structural-functionalism. Many scholars have likewise found that the Chinese government extensively employs post removal, erasure of collective memory, and social media bots to safeguard national security, social stability, and regime legitimacy. This cancellation culture is far more covert, secure, and effective at avoiding public resistance than explicit command-and-prohibit measures.

The ambivalence of state presence is a central theme in debates on digital authoritarianism. Xue and Van Stekelenburg (2018), for example, argue that China’s Internet has simply shifted civic behavior online under strict monitoring–making the state’s job of curbing activism easier. In their view, online channels offer the illusion of greater expression (like hashtag politics) while the government simultaneously escalates censorship. Our results partly confirm this: high state visibility did suppress participation on average. But the flip side is that when digital environments are saturated with support and shared grievances, state surveillance can act like a catalyst. This echoes what Mertha (2009) calls “fragmented authoritarianism 2.0”: the policy process remains controlled by the Party-state, but channels for grievance redress (including online campaigns and petitions) have expanded. In practice, this means that some officials rely on the Internet to scan public sentiment, tolerating expressions that were once directed at local leaders. Thus, under certain conditions of social solidarity, perceived state oversight may not purely intimidate but can inadvertently delegate a stage for articulation of demands.

Another finding is that State power does not invariably play a negative role in citizens’ political participation. Data analysis revealed that the stronger the perception of State Presence, the greater the effect of Social Support on Online Political Participation via Digital Media Use. A key underpinning of this moderating effect is that, when controlling for perceived State Presence, Social Support via Digital Media Use actually suppresses youth Online Political Participation. One possible explanation is that perceived Social Support in this model is closely related to trust in government and political efficacy. Furthermore, the anonymity afforded by Online Political Participation substantially reduces the risk associated with non-institutionalized engagement for ordinary citizens. As a risk-taking behavior, Online Political Participation is influenced not only by situational awareness and risk perception but also by individual trust (Mayer et al., 1995). Many scholars have found that lower public trust in government corresponds with a greater propensity to adopt lower-risk forms of political participation, such as Online Political Participation via digital platforms (Ceron, 2015).

In the Chinese setting, this rethinking is particularly important. Offline protest is heavily restricted, so digital expression often *is* the only outlet. Moreover, the content of online acts can carry subtle political meaning. As Yang and Jiang (2015) show, posting satirical images or jokes may not overtly challenge the state, yet these acts can “drive” officials to respond to an issue indirectly. Similarly, collective rituals like online mourning (e.g., the Li Wenliang case) have been documented as mobilizing tools under strict censorship. Thus, a binary view that hashtags and likes are trivial misses their strategic role in China’s “managed activism.” Our data align with this: even in heavily moderated forums, youth use coded language and creative symbols to express support or grievance. By measuring a broad typology – petition, activism, and expression – we capture this nuance. We echo Theocharis et al. in urging that political communication research formally integrate these digital behaviors into its concept of participation. At the same time, the climate of surveillance calls for caution. We must recognize that not all online participation is equally effective or free of risk. The “digital citizenship” of Chinese youth often involves a balancing act: using official channels to air concerns (which the regime tolerates) while hunting for subversive cues under the radar. This hybridity is a blind spot of many Western models, which assume either open deliberation or outright repression. In reality, Chinese youth navigate a *continuum* of affordances and limits. Our findings suggest that models of online participation should account for this fluidity – for instance, by incorporating perceived risk into measures of political efficacy.

Finally, our study reinforces a growing consensus that the “clicktivism” critique (the idea that online civic acts are superficial) is insufficient, especially in the Chinese context. Traditionally, political communication scholars have discounted online participation as mere leisure (Hirzalla and Zoonen, 2011; Gibson and Cantijoch, 2013). However, recent theory insists that “digitally networked participation” itself constitutes political engagement. Theocharis et al. (2021) argue that activities like sharing a petition, liking an environmental campaign, or posting a meme should be counted as political, because these acts contribute to collective political awareness and can lead to offline action. Our findings support this perspective: even our “expressive” measures (like posting a commentary) significantly correlated with more traditional forms (like signing petitions) once social support and media use are accounted for. In other words, Chinese youth do not neatly separate offline activism from online expression; rather, small online acts (amplified by social networks) appear to knit together broader participation. We conceptualized “online political participation” not as a single behavior, but through multiple indices: expressive acts (sharing or commenting on political content), targeted activism (e.g., online campaigning, organizing virtual groups), and formal petitioning (sharing or signing online petitions). This mirrors the approach of recent scholars who insist on capturing both conventional and creative digital acts. For example, respondents who engaged in expressive posting – even sharing political memes or satire – were more likely to also engage in activism when their social network use was high. Prior work (Yang and Jiang, 2015) shows that such expressive behaviors may not directly seek to influence officials but can “indirectly influence government” by shaping public mood. In sum, our study underscores that, in contemporary China, online political participation is best understood as a *spectrum* of behaviors that are tightly scaffolded by social support and mediated through digital channels.

### 6.1 Limitations

This study focuses on youth from a specific Chinese province, and the data were collected in 2018 as cross-sectional, static observations, precluding dynamic trend analysis. Although the large sample provides a comprehensive snapshot of Chinese youths’ digital media use, real-world social support, and political participation, the post-COVID-19 context may have altered both the forms and content of youth political participation. Whether these actions have changed under higher digital media use and constrained offline participation remains a topic for future research. Furthermore, during the COVID-19 pandemic, the State intensified its panoramic surveillance of digital spaces and suppressed individual political agency to eliminate potential resistance to lockdown policies. The chilling effect then permeated society at large. The use of pre-pandemic data in this study is less about reconstructing a tranquil past before the crisis than about forecasting and anticipating a future in which individual agency and prosocial attitudes are restored.

## Data Availability

The datasets presented in this study can be found in online repositories. The names of the repository/repositories and accession number(s) can be found in the article/Supplementary material.
