# Supplementary material for: Resilience under the chilling effect: how social support and digital media reshape online political participation among Chinese youth
Source: Front Psychol. 2025 Aug 4;16:1634604. doi: 10.3389/fpsyg.2025.1634604 (PMC12358391; doi:10.3389/fpsyg.2025.1634604)
Supplement: Supplementary file 1 [file Table_1.docx]

Supplementary Material

**INFORMED CONSENT FORM**

You are invited to participate in an on/offline activities study​ conducted by ​​researchers ​. Participation is voluntary, and you may withdraw anytime without penalty. The questionnaire includes approximately ​​15​​ questions (estimated time: 30 minutes​) on topics such as media use​, on/offline activities.

This study involves no direct physical or psychological risks, though you may skip questions or withdraw at any time. Your anonymous responses will be collected by researchers. All data will be used ​exclusively in aggregated form for academic publication and will not be shared with commercial entities or used for profit-driven activities.\

To withdraw or request data deletion (valid within 7 days post-submission), contact ​​[email]​. For research inquiries: ​​[Name, Email, Phone]​; for ethical concerns: ​​[Ethics Committee, Email, Phone]​.

**Survey Questionnaire**

I. Demographics

1. Gender: (1) Male (2) Female

2. Age: _______

3. Marital status: (1) Married (2) Single (3) Divorced (4) Widowed

4. Are you an only child? (1) Yes (2) No

5. Education level: (1) Primary school or below (2) Junior secondary (3) Senior secondary/technical/vocational (4) Junior college (5) Bachelor’s degree (6) Graduate degree or above

6. Hukou status (single choice): (1) Local urban (2) Local rural (3) Non-local urban (4) Non-local rural

7. Living arrangement: (1) Living alone (2) Sharing with others (e.g., roommates, dormitory) (3) Living with parents/elders (4) Living with spouse and children (5) Living with parents, spouse, and children

8. Occupation (single choice):
(1) Civil servant (2) Public institution staff (3) Private enterprise staff (including self-employed) (4) State-owned or state-controlled enterprise staff (5) Staff of foreign- or Hong Kong/Macau/Taiwan-invested enterprises (6) Heads of government agencies, party and mass organizations, enterprises, or public institutions (7) Professional/technical personnel (e.g., lawyers, finance, medical, IT) (8) University faculty/researchers (9) Commerce/service workers (10) Agricultural, forestry, animal husbandry, fishery, or water conservancy workers (11) Military personnel (12) Migrant workers/production or transport equipment operators and related staff (13) Freelancers (14) Full-time students (15) Other: _________

9. Personal average monthly income (RMB):
(1) No fixed income (2) ≤ 1,000 (3) 1,001–3,000 (4) 3,001–5,000 (5) 5,001–7,000 (6) 7,001–10,000 (7) 10,001–20,000 (8) 20,001–30,000 (9) ≥ 30,001

10. Please rate your current social status on a scale from 1 (perceived lowest) to 10 (perceived highest).

11. Please rate your social status five years ago on a scale from 1 to 10.

12. How frequently do you engage in the following online activities?

|  | **Never** | **Rarely** | **Occasionally** | **Sometimes** | **Often** |
| --- | --- | --- | --- | --- | --- |
| Comment on news articles/news portal websites online | 1 | 2 | 3 | 4 | 5 |
| Publicly express opinions or publish information about social/public affairs online | 1 | 2 | 3 | 4 | 5 |
| Repost text, videos, and images related to social/public affairs online | 1 | 2 | 3 | 4 | 5 |
| Participate in online discussions about social/public affairs | 1 | 2 | 3 | 4 | 5 |
| Post online (e.g., on Weibo/social media) seeking help when encountering difficulties or social injustice | 1 | 2 | 3 | 4 | 5 |
| Seek help from social media influencers when encountering difficulties or injustice | 1 | 2 | 3 | 4 | 5 |
| Organize or participate in collective actions or protests concerning social/public affairs | 1 | 2 | 3 | 4 | 5 |
| Participate in actions like online donations | 1 | 2 | 3 | 4 | 5 |
| Contact government departments regarding social/public affairs | 1 | 2 | 3 | 4 | 5 |
| Provide feedback to People's Congress deputies or CPPCC members concerning social/public affairs | 1 | 2 | 3 | 4 | 5 |
| Contact newspapers, radio stations, or TV stations regarding social/public affairs | 1 | 2 | 3 | 4 | 5 |
| Join groups or online communities related to social/public affairs | 1 | 2 | 3 | 4 | 5 |

13. For the following statements regarding the socialist core values and dissemination of mainstream ideology, please rate your agreement on a 1–5 scale.

|  | **Strongly Disagree** | **Disagree** | **Neutral** | **Agree** | **Strongly Agree** |
| --- | --- | --- | --- | --- | --- |
| I am well acquainted with the content and fundamental tenets of the socialist core values. | 1 | 2 | 3 | 4 | 5 |
| I frequently encounter state‑led ideological messaging. | 1 | 2 | 3 | 4 | 5 |
| The State should proactively lead the construction of a unified mainstream ideology and values. | 1 | 2 | 3 | 4 | 5 |
| The State should strengthen Internet information governance. | 1 | 2 | 3 | 4 | 5 |
| The State should enhance the promotion of mainstream ideology online. | 1 | 2 | 3 | 4 | 5 |

14. To what extent do you agree with the following statements?

|  | Very dissatisfied | Dissatisfied | Neutral | Satisfied | Very satisfied |
| --- | --- | --- | --- | --- | --- |
| The adequacy of basic public infrastructure in your city | 1 | 2 | 3 | 4 | 5 |
| The extent of policy support for youth development in your city | 1 | 2 | 3 | 4 | 5 |
| The availability of social services for youth in your city | 1 | 2 | 3 | 4 | 5 |
| The safety of the developmental environment for the next generation in your city | 1 | 2 | 3 | 4 | 5 |
| The harmony and quality of your own family environment | 1 | 2 | 3 | 4 | 5 |
| The friendliness of your community environment and neighborhood relationships | 1 | 2 | 3 | 4 | 5 |
| I believe the society in which I live is fair. | 1 | 2 | 3 | 4 | 5 |
| I am satisfied with my current life and consider myself happy. | 1 | 2 | 3 | 4 | 5 |
| Most people in this society are trustworthy. | 1 | 2 | 3 | 4 | 5 |
| I believe the social environment in which I live is very safe. | 1 | 2 | 3 | 4 | 5 |

15. How frequently do you engage in the following activities or use the following platforms?

|  | Never | Rarely | Occasionally | Sometimes | Frequently |
| --- | --- | --- | --- | --- | --- |
| Monitoring major policy announcements in your locality via online media, social media, or portals. | 1 | 2 | 3 | 4 | 5 |
| Following trends and commentary on local socio‑economic development via online media, social media, or portals. | 1 | 2 | 3 | 4 | 5 |
| Accessing online resources related to public affairs. | 1 | 2 | 3 | 4 | 5 |
| Following news events on television, newspapers, magazines, or radio. | 1 | 2 | 3 | 4 | 5 |
| WeChat | 1 | 2 | 3 | 4 | 5 |
| Sina Weibo | 1 | 2 | 3 | 4 | 5 |
| Zhihu | 1 | 2 | 3 | 4 | 5 |
| News platforms  (e.g., Toutiao) | 1 | 2 | 3 | 4 | 5 |
| Short-video social platforms  (e.g., Douyin, Kuaishou) | 1 | 2 | 3 | 4 | 5 |
| Video-sharing websites  (e.g., Youku, Bilibili) | 1 | 2 | 3 | 4 | 5 |
| Audio platforms  (e.g., Ximalaya) | 1 | 2 | 3 | 4 | 5 |
